# γδ T Cells’ Role in Donor-Specific Antibody Generation: Insights From Transplant Recipients and Experimental Models

**DOI:** 10.3389/ti.2025.12859

**Published:** 2025-01-29

**Authors:** Xavier Charmetant, Guillaume Rigault, Chien-Chia Chen, Hannah Kaminski, Jonathan Visentin, Benjamin Taton, Gabriel Marseres, Virginie Mathias, Alice Koenig, Thomas Barba, Pierre Merville, Stéphanie Graff-Dubois, Emmanuel Morelon, Julie Déchanet-Merville, Valérie Dubois, Jean-Paul Duong van Huyen, Lionel Couzi, Olivier Thaunat

**Affiliations:** ^1^ Centre International de Recherche en Infectiologie, INSERM U1111, Université Claude Bernard Lyon I, CNRS UMR5308, Ecole Normale Supérieure de Lyon, Univ. Lyon, Lyon, France; ^2^ Department of Transplantation, Nephrology and Clinical Immunology, Edouard Herriot Hospital, Hospices Civils de Lyon, Lyon, France; ^3^ Lyon-Est Faculty of Medicine, Claude Bernard University (Lyon 1), Villeurbanne, France; ^4^ Department of Surgery, National Taiwan University Hospital, Taipei, Taiwan; ^5^ Department of Nephrology, Transplantation, Dialysis and Apheresis, Bordeaux University Hospital, Bordeaux, France; ^6^ ImmunoConcEpT, CNRS, Université de Bordeaux, UMR 5164, Bordeaux, France; ^7^ Laboratory of Immunology et Immunogenetics, Pellegrin Hospital, Bordeaux, France; ^8^ French National Blood Service (EFS), HLA Laboratory, Décines, France; ^9^ Department of Internal Medicine, Edouard Herriot Hospital, Hospices Civils de Lyon, Lyon, France; ^10^ Sorbonne Université, INSERM, UMRS 959, Immunology-Immunopathology-Immunotherapy (i3), Paris, France; ^11^ Pathology Department, Necker Hospital, Assistance Publique-Hôpitaux de Paris, Paris, France

**Keywords:** humoral response, translational science, gamma delta T cell, donor specific antibody (DSA), B cell

## Abstract

The generation of donor-specific antibodies (DSA) requires that alloreactive B cells receive help from follicular helper T (T_FH_) cells. Recent works have suggested that γδ T cells could contribute to T cell-dependent humoral responses, leading us to investigate their role in DSA generation. Analysis of a cohort of 331 kidney transplant recipients found no relation between the number of circulating γδ T cells and the risk to develop DSA. Coculture models demonstrated that activated γδ T cells were unable to promote the differentiation of B cells into plasma cells, ruling out that they can be “surrogate” T_FH_. In line with this, γδ T cells preferentially localized outside the B cell follicles, in the T cell area of lymph nodes, suggesting that they could instead act as “antigen-presenting cell” (APC) to prime αβ T_FH_. This hypothesis was proven wrong since γδ T cells failed to acquire APC functions *in vitro*. These findings were validated *in vivo* by the demonstration that following transplantation with an allogeneic Balb/c (H2^d^) heart, wild-type and TCRδKO C57BL/6 (H2^b^) mice developed similar DSA responses, whereas TCRαKO recipients did not develop DSA. We concluded that the generation of DSA is unfazed by the absence of γδ T cells.

## Graphical Abstract

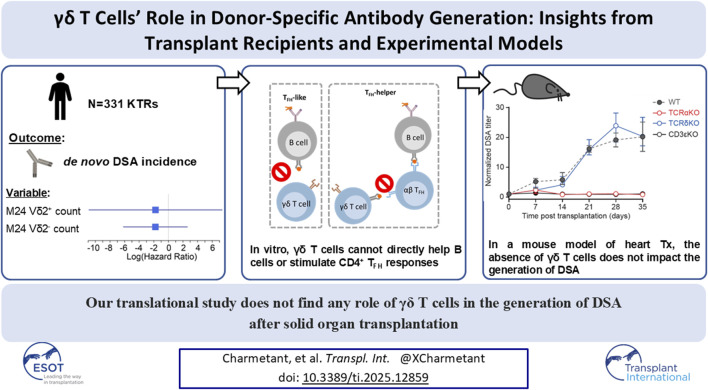

## Introduction

Despite the progress in therapeutic immunosuppression, 10%–20% of graft recipients develop *de novo* alloantibodies directed against donor-specific alloantigens (donor specific antibodies, DSA) within 5 years post-transplantation [1, 2].

DSA are produced by recipient’s plasma cells located in the bone marrow and the spleen [3] and released in the circulation, in which they remain sequestrated due to their size [4]. Once bound to the only accessible allogeneic HLA molecules of graft endothelium [4], DSA can activate the classical complement pathway, and/or recruit innate effectors through surface Fc receptors [5, 6]. These two mechanisms are responsible for the microvascular inflammation that is characteristic of antibody-mediated rejection (AMR) [5]. In absence of efficient curative treatment for AMR, the latter is recognized as the main cause of allograft loss [7, 8] and prevention of *de novo* DSA appears therefore as the best prospect to prolong graft survival.

The current immunologic dogma holds that *de novo* DSA generation is initiated in recipient’s secondary lymphoid organs [9], and depends upon a T-cell dependent humoral response, which implies that recipient’s B cells need to receive help from either recipient’s CD4^+^ αβ T cells [i.e., the canonical indirect pathway of allorecognition [10–12]], or from the CD4^+^ αβ T cells of donor origin that were present within the graft at the time of procurement [i.e., the more recently described inverted direct pathway [12–14]].

Alongside αβ T cells, another subset of lymphocytes that expresses a γδ TCR has long been described [15]. Despite a growing interest in the field of transplantation for γδ T cells [16], the role of this immune subset in DSA generation has never been explored so far. γδ T cells are equipped with a clonally rearranged TCR, which is usually not restricted to classical MHC molecules but instead directly recognizes phospho-antigens [17–19] or stress-induced antigens [20, 21]. In response to stimulation through their TCR and/or natural killer receptors [22] or toll-like receptors [23, 24], γδ T cells are capable of cytotoxicity and cytokine secretion that participate in innate responses against pathogens [25, 26] and cancer [22].

A recent study has however demonstrated that γδ T cells recognizing tumor antigen in an HLA-I restricted manner could be generated *in vitro* and identified in the normal human repertoire [27] suggesting that, in addition to their innate functions, γδ T cells could also be involved in adaptive immune responses. Several experimental studies have reported that γδ T cells can promote humoral responses, either by directly supporting the germinal center reaction and switched antibody responses [T_FH_-like function; [28, 29]], or by presenting the antigen to CD4^+^ T cells and orienting their differentiation into T_FH_ [T_FH_-helper function; [30]]. Based on this literature, we put forward the hypothesis that γδ T cells may be involved in the generation of DSA after solid organ transplantation and used a translational approach to rigorously test the validity of this theory.

## Materials and Methods

### Flow Cytometry Analyses for the Monitoring of γδ T Cells

Kidney transplant recipients were followed for > 2 years post-transplantation with peripheral blood immunophenotyping and serological follow-up. Vδ2^−^ and Vδ2^+^ γδ T cells counts were obtained by flow cytometry at day 0 and 2 years post-transplantation. For immunophenotyping, >5,000 lymphocytes were stained with anti-CD45, antipan-δ (clone IMMU 510; Beckman Coulter, Krefeld, Germany), and anti-TCR Vδ2 (clone 15D; Thermo Fisher Scientific, Rockford, IL). Percentages were obtained using CELLQUEST software (BD Bioscience), and absolute counts with the Single–Platform Lyse/No–Wash Trucount (BD Bioscience).

### Anti-HLA Antibody Detection and Characterization

Sera samples were analyzed using Single-antigen Bead Assay (One Lambda, Canoga Park, CA). Only DSA with MFI >500 were considered.

### Lymph Node Histology

Samples are normal, tumor-free peripheral lymph nodes, obtained from cancer excision surgery. Formalin-fixed paraffin-embedded (FFPE) sections were stained with an automat (LEICA BOND-III, Leica Biosystems) using anti-human TCRβ (anti-T-cell receptor [TCR]β antibody; clone G11; Santa Cruz Biotechnology) and TCRδ (anti–T-cell receptor [TCR]δ antibody; clone H41; Santa Cruz Biotechnology) mAbs. Computer-assisted morphometric quantifications were performed using FIJI software [31].

### γδ T Cell Activation

Human Peripheral Blood Mononuclear Cells (PBMC) were collected from healthy volunteers and isolated by centrifugation on a Ficoll density gradient. Human splenocytes were collected from deceased organ donors.

Two million cells were cultured overnight in 500 µL of complete medium [RPMI 1640 GlutaMAX medium (Invitrogen) supplemented with 10% fetal calf serum, 25 mM Hepes (Invitrogen), and penicillin/streptomycin (10 U/mL; Invitrogen)] at 37°C and 5% CO_2_, with or without Dynabeads^TM^ Human T-activator CD3/CD28 (ThermoFisher Scientific, 1 Dynabead for 1 PBMC), IL-18 (50 ng/mL, PreproTech) or IL-2 (100 IU/mL, R&D Systems) + IL-15 (10 ng/mL, PreproTech). In some conditions, anti-CD40L (clone TRAP1, BD Biosciences) antibody was added to the culture medium (10 µL per condition). After removal of the Dynabeads, cells were incubated at 4°C with relevant antibodies: CD3 (clone UCHT1, BD Biosciences), CD4 (clone SK3, BD Biosciences), TCRγδ (clone REA-591, Miltenyi Biotec), Vδ2 (clone REA-771, Miltenyi Biotec), CD19 (clone HIB19, BD Biosciences), CXCR5 (clone RF8B2, BD Biosciences), CD69 (clone FN50, BD Biosciences), MHC-II (clone G46-6, BD Biosciences), CD80 (clone 2D10, Biolegend), CD86 (clone FUN-1, BD Biosciences), and a fixable viability dye (ThermoFisher Scientific). Samples were acquired on a BD LSRFortessa flow cytometer (BD Biosciences). Data were analyzed with FlowJo software (Tree Star).

### Cocultures

B cells, CD4^+^ and γδ T cells were purified from PBMCs (95% purity) by negative selection kits (Stemcell). B cells were stained with CellTrace Violet (ThermoFisher Scientific). 4 × 10^4^ B cells were cocultured either with 4 × 10^5^ allogeneic CD4^+^ T cells or 3.2 × 10^5^ allogeneic CD4^+^ T cells plus 8 × 10^4^ syngeneic γδ T cells. A soluble anti-human IgM F (ab’)_2_ (5 μg/mL, Jackson Immunoresearch) was added to the culture medium. After 6 days, cells were stained with fluorescent antibodies directed against: CD3 (clone UHCT1), CD4 (clone SK3), CD19 (clone HIB19), CD20 (clone 2H7), all from BD Biosciences, and a Fixable Viability Dye (eBiosciences). Sample acquisitions were made on a BD LSR Fortessa flow cytometer (BD Biosciences). Data were analyzed with FlowJo software (Tree Star).

### Mice

Wild-type C57BL/6 (H-2^b^) mice and wild-type or nude Balb/c (H-2^d^) mice were purchased from Charles River Laboratories (Saint Germain sur l’Arbresle, France). TCR α [32] on C57BL/6 genetic background (TCRαKO) were obtained from the Centre de Distribution, Typage et Archivage animal (Orléans, France). TCR δ knock out [33] mice on C57BL/6 genetic background (TCRδKO) were provided by B. Malissen. CD3εKO mice on C57BL/6 genetic background were purchased from The Jackson Laboratory (Bar Harbor, ME, United States).

All mice were maintained under EOPS conditions in our animal facility^1^.

### Heterotopic Heart Transplantation

Murine heterotopic heart transplantations were performed as previously described [4, 11, 34]. Briefly, cardiac allografts were transplanted into subcutaneous space of right neck. Anastomoses were performed by connecting end-to-end the ascending aorta of the graft with the recipient’s common carotid artery and by pulling the main pulmonary artery with the external jugular vein. DSA titer was determined using a custom flow cross match assay ([4, 11], Supplementary Methods).

### Statistical Analysis

All the analyses were performed using R software version 4.2.0 (R Foundation for Statistical Computing; 2021;^2^) and/or GraphPad Prism v8.0. Quantitative variables were expressed as median ± IQR and compared using Mann-Whitney test when two groups were compared, Kruskal-Wallis test when more than two groups were compared, and two-way ANOVA when there was a within-group comparison between two different conditions. All tests were two-sided. Cox regression was used to assess the relationship between the numbers of circulating Vδ2+ or Vδ2- γδ T cells and the incidence of *de novo* DSA.

Statistical significance was considered for a p-value <0.05.

### Ethic

The study was carried out in accordance with French legislation on biomedical research and the Declaration of Helsinki. All patients gave written informed consent for the utilization of clinical data and biological samples for research purpose (CNIL final agreement, decision 2009-413, no. 1357154).

Human spleen samples were used in accordance with the authorization issued by the French Ministry of Higher Education, Research and Innovation (authorization AC 2020-3959).

Studies and procedures in mice were performed in accordance with EU guidelines and were approved by the local ethical committee for animal research (CECCAPP: #C2EA15).

## Results

### Higher Numbers of Circulating γδ T Cells Do Not Correlate With an Increased Risk for *De Novo* DSA

In humans, γδ T cells are divided into two subsets. The Vδ2 chain associates preferentially with the Vγ9 chain, resulting in the Vδ2^+^Vγ9^+^ (hereafter referred to as Vδ2^+^) subpopulation. These cells are activated by endogenous or bacterial phosphoantigens in a butyrophilin-dependent manner [17–19]. The other group of γδ T cells mainly encompasses Vδ1^+^ or Vδ3^+^ cells (hereafter referred to as Vδ2^−^) and is thought to be sensitive to a broad panel of stress-induced antigens [20, 21].

**FIGURE 1:**
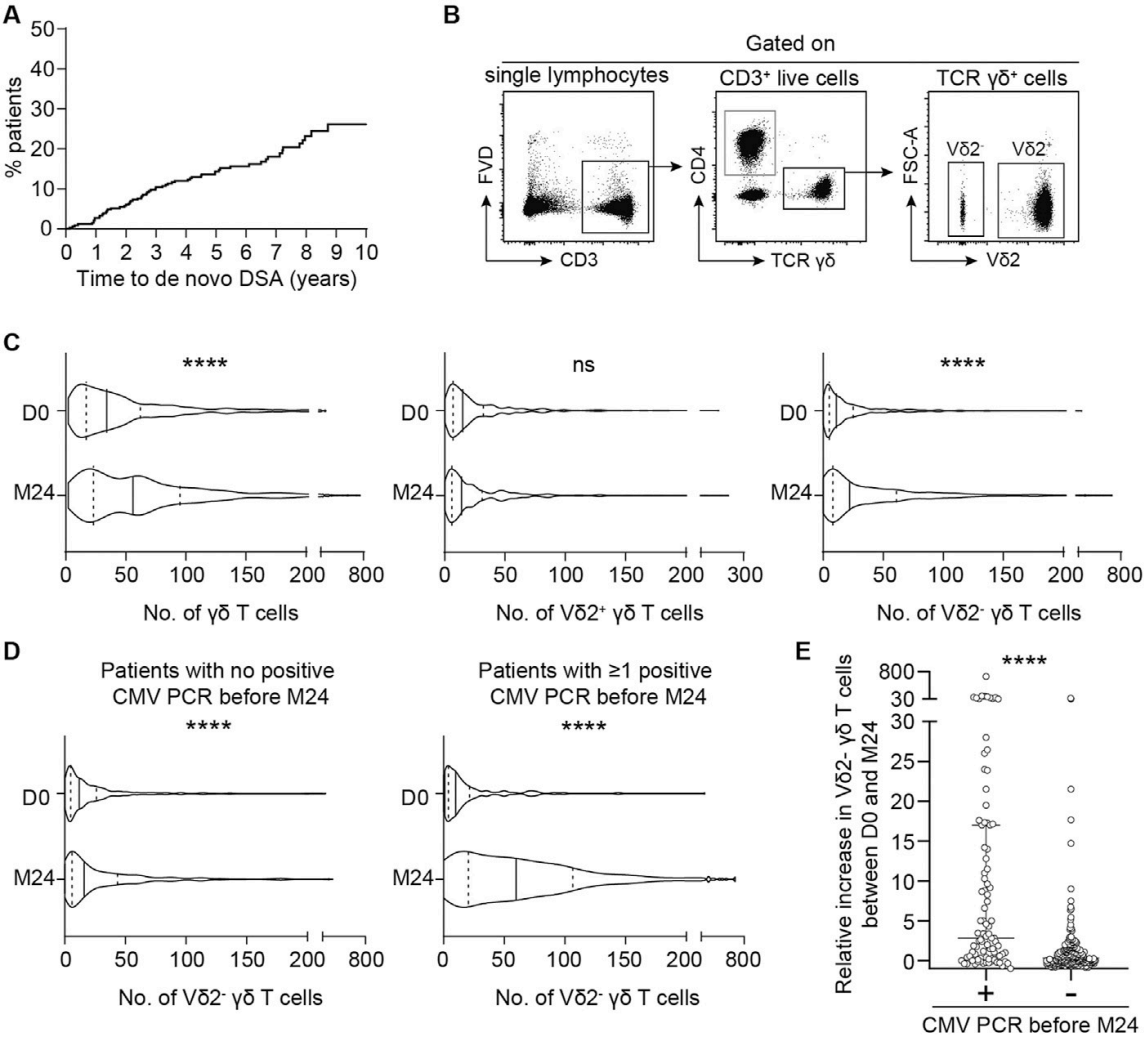
Serological and cellular follow-up of kidney transplant recipients. **(A)** Kaplan-Meier curve of DSA incidence after kidney transplantation in the cohort. **(B, C)** Kidney transplant recipients T cells were phenotyped by flow cytometry the day of the transplantation (D0) and 2 years later (M24). **(B)** Representative flow cytometry profiles of the gating strategy used to assess the numbers of the different γδ T cells subtypes. **(C)** The number of total γδ T cells (left panel), Vδ2^+^ γδ T cells (middle panel) or Vδ2^−^ γδ T cells (right panel) measured on the day of the transplantation (D0) and M24 were compared. The median (solid line) and the 25th and 75th percentiles (dotted lines) are represented. Mann-Whitney test, ****P < 0.0001. **(D)** The number of Vδ2^−^ γδ T cells measured on the day of the transplantation (D0) and M24 were compared in patients with no positive CMV PCR before M24 (left panel) and in patients with ≥ 1 positive CMV PCR before M24 (right panel). The median (solid line) and the 25th and 75th percentiles (dotted lines) are represented. Mann-Whitney test, ****P < 0.0001. **(E)** Relative increase in Vδ2^−^ γδ T cells between D0 and M24 in patients with or without CMV PCR positivity during the first 2 years. Mann-Whitney test, ****P < 0.0001.

To assess the potential involvement of the two subsets of γδ T cells in DSA generation, we took advantage of a cohort of 331 kidney transplant recipients (KTRs) that did not receive a depleting induction and for whom the γδ T cell populations and DSA had been prospectively monitored during a 10 years follow-up period (Supplementary Figure S1). The main clinical characteristics of the cohort are presented in Supplementary Table S1. Sixty-two KTRs (18.7%) developed *de novo* DSA during the follow-up period (Figure 1A). The numbers of circulating Vδ2^+^ and Vδ2^−^ γδ T cells were measured by flow cytometry at baseline and 2 years after transplantation (Figure 1B). Overall, the total number of γδ T cells significantly increased between the day of the transplantation and 2 years post-transplantation (Figure 1C). This was explained by the expansion of the Vδ2^−^ subset in response to CMV replication during the first 2 years (Figure 1D and references [25, 35, 36]). Indeed, even if patients without detectable DNAemia increased their absolute numbers of Vδ2^−^ T cells, the relative increase was much more important after CMV viremia (median relative increase of 2.833 versus 0.3333 in CMV DNA-positive versus CMV DNA-negative groups, respectively; Mann Whitney test, p < 0.0001; Figure 1E). Finally, if we define an expansion of the Vδ2^-^ population as a relative increase of more than 1.1, patients with this expansion exhibited a significantly higher incidence of CMV viremia (Chi-square test, p < 0.0001).

To assess whether the circulating levels of Vδ2^+^ or Vδ2^-^ γδ T cells at the time of transplantation influenced the development of *de novo* DSA 2 and 10 years post-transplantation, we performed two Cox regression analyses. The results showed no significant association (Table 1). However, given that the incidence of DSA was stable over the follow-up period on one hand (Figure 1A), most CMV infections occur in the 1st year after transplantation [37, 38], and the pool of expanded Vδ2^-^ γδ T cells remains stable over time [25] on the other, we performed a third Cox analysis to assess the relation between the number of circulating Vδ2^+^ or Vδ2^-^ γδ T cells at 2 years and the risk to develop DSA from 2 to 10 years post-transplantation. KTRs who developed DSA before 2 years (n = 22) were therefore excluded from this analysis. Once again, we found no association between the number of circulating Vδ2^+^ or Vδ2^-^ γδ T cells at month 24 and the incidence of *de novo* DSA between 2 and 10 years post-transplantation (Table 1).

**TABLE 1:** Results of the Cox regression model used to assess the role of Vδ2^+^ or Vδ2^−^ on *de novo* DSA incidence.

Model	Variable		Outcome	
		10-years DSA incidence		
		HR	95% CI	p value
1	D0 Vδ2^+^ count	6.690	[0.003682; 12156]	0.620
	D0 Vδ2^−^ count	9.036	[0.004701; 17368]	0.568
2	M24 Vδ2^+^ count	0.1704	[1.855e-05; 1565.3]	0.704
	M24 Vδ2^−^ count	0. 1682	[2.097e-03; 13.5]	0.426
		**2-years DSA incidence**		
3	D0 Vδ2^+^ count	12.897	[3.609e-05; 4609646]	0.695
	D0 Vδ2^−^ count	218.744	[6.390e-03; 7488249]	0.312

### Evaluation of T Follicular Helper-Like Function of Human γδ T Cells

**FIGURE 2:**
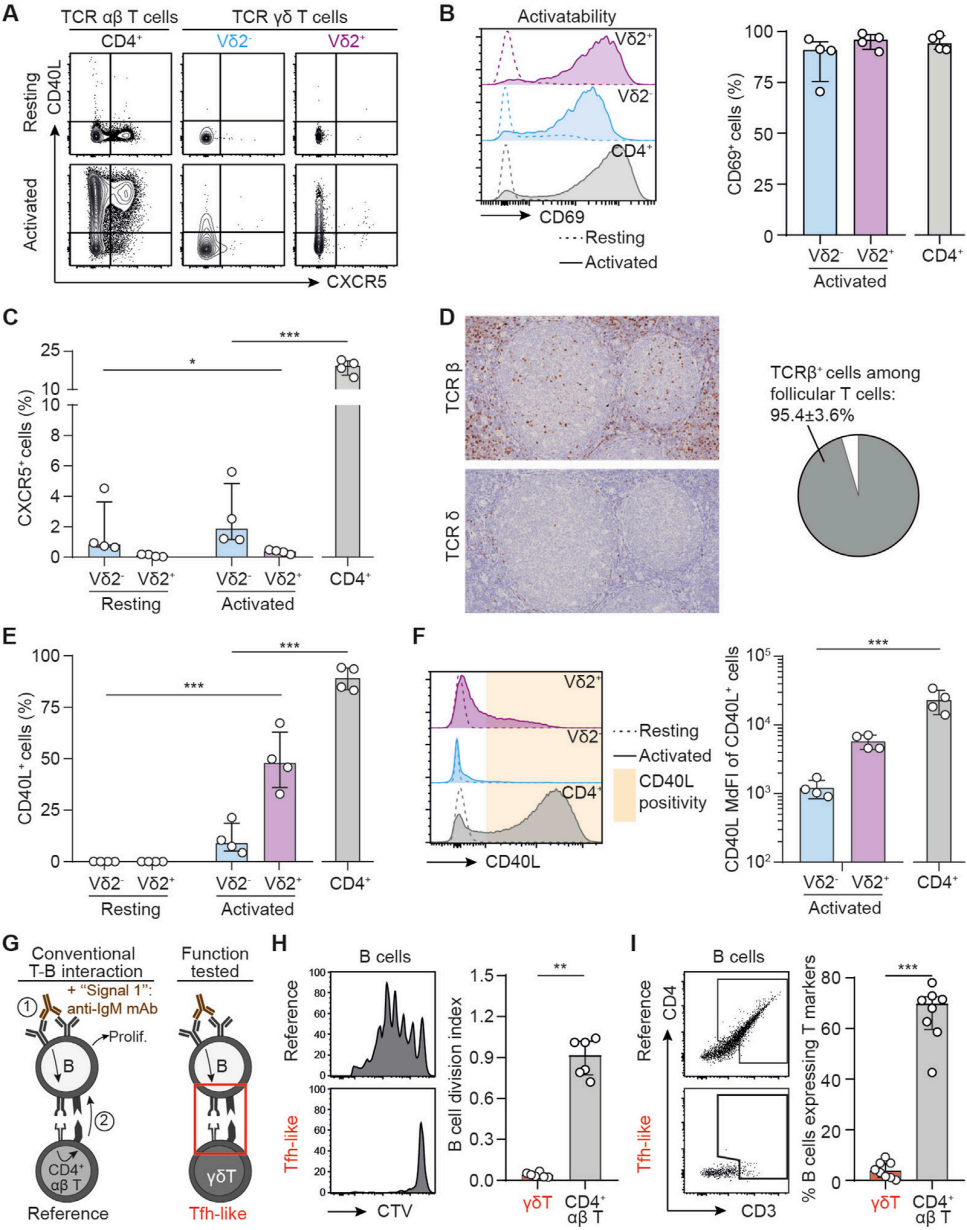
T follicular helper-like function of human γδ T cells. **(A–C)** PBMCs were cultured in the presence or absence of beads coated with anti-CD3 and anti-CD28 mAbs. **(A)** Representative flow cytometry profiles for the expression of CD40L and CXCR5 in resting (upper row) and activated (lower row) T cells. **(B)** Left: Representative histograms for the expression of CD69 in resting (dotted line) or activated (full line) Vδ2^+^ (up, purple), Vδ2^−^ (middle, blue) or control CD4^+^ αβ T cells (down, grey). Right: individual values for percentages of CD69^+^ cells. **(C)** Individual values for percentages of CXCR5^+^ cells. **(D)** Left: immunohistochemical sections of a human lymph node, stained for TCRβ (upper thumbnail) and TCRδ (lower thumbnail). Right: pie-chart representing the proportion of TCRβ^+^ and TCRδ^+^ cells among follicular T cells after quantification by computer-assisted morphometry. **(E)** Individual values for percentages of CD40L^+^ cells. **(F)** Left: Representative histograms for the expression of CD40L in resting (dotted line) or activated (full line) Vδ2^+^ (up, purple), Vδ2^−^ (middle, blue) or control CD4^+^ αβ T cells (down, grey). Right: individual MdFI values for CD40L^+^ cells. **(G–I)** Human B cells were cocultured with allogeneic CD4^+^ T or γδ T cells in the presence of IgM F(ab′)2 (signal 1), and **(H)** the percentage of divided cells among alive B cells was evaluated by flow cytometry, as well as **(I)** the trogocytosis between B and T cells. **(G)** Schematic representation of the experiment. **(H)** Left: Representative histograms. Right: Individual coculture values. **(I)** Left: The flow cytometry gating strategy for the assessment of trogocytosis. Right: percentage of B cells that have experienced trogocytosis in each coculture. Data are presented as median ± IQR. Data were analyzed by Mann-Whitney test when two groups were compared, Kruskal-Wallis test when more than two groups were compared, and two-way ANOVA when there was a within-group comparison between two different conditions. *P < 0.05, **P < 0.01 and ***P < 0.001.

To test if human γδ T cells can act as surrogate follicular helper T cells (T_FH_) and support the differentiation of allospecific B cells into DSA-producing plasma cells, peripheral blood mononuclear cells (PBMCs) from 4 healthy volunteers were cultured with or without beads coated with anti-CD3 and anti-CD28 mAbs. The expression of CXCR5 [a chemokine receptor allowing T_FH_ cell migration towards B-cell area in secondary lymphoid organs, [39]] and CD40L [a key costimulatory molecule for B cells responses to T cell-dependent antigens, [40]] was assessed by flow cytometry at the end of overnight cultures (Figure 2A). CD4^+^ αβ T cells, which encompass T_FH,_ the subset specialized in providing help to B cells, were used as reference. A prerequisite for drawing conclusions about activation-induced phenotypic modification, was to demonstrate that all 3 subsets had the same capacity to respond to the *in vitro* stimulation. In line with this, we observed that the 3 T cell subsets upregulated the surface activation marker CD69 similarly upon *in vitro* stimulation (Figure 2B). The expression of CXCR5 by γδ T cells was barely detectable at steady state and did not increase after activation, whereas a median of 19.3% (IQR 15.6–21.2) of CD4^+^ αβ T cells expressed CXCR5 after activation (Figure 2C). If γδ T cells do not express CXCR5 to a significant degree [an observation also made by other independent groups [41]], in theory they should not be found in the secondary follicles of secondary lymphoid organs. To confirm this hypothesis, normal human lymph nodes [i.e., a site where the alloimmune response takes place after transplantation [9]] were stained with either an anti-TCRβ or an anti-TCRδ antibody and the spatial distribution of γδ T cells was compared to that of αβ T cells, the subset of T cells providing canonical help to B cells. The density of TCRβ^+^ cells in the secondary follicles (i.e., the germinal centers) was much higher than that of TCRδ^+^ cells. As a consequence, TCRβ^+^ cells represent around 95% of T cells in the germinal centers, even if some rare TCRδ^+^ could be found in some follicles (Figure 2D).

Interestingly, and in line with previous works [29], the Vδ2^+^ subset (but not Vδ2^−^) was able to significantly upregulate CD40L expression after activation, albeit in lower proportion than CD4^+^ αβ T cells (47.8%, IQR 36.1 to 62.3 versus 89.2%, IQR 83.6 to 94.1, p = 0.0286; Figure 2E). Furthermore, the level of expression of CD40L (assessed by the median fluorescence intensity, MdFI) of CD40L^+^ Vδ2^+^ T cells tend to remain lower than that of CD40L^+^ CD4^+^ αβ T cells (5688, IQR 4596 to 7037 versus 21861, IQR 15139 to 32121, Figure 2F). Finally, it should be noted that the γδ subset that expresses CD40L the most (Vδ2^+^ cells) is those with the lowest ability to upregulate the expression of CXCR5, making unlikely that Vδ2^+^ cells could act as surrogate T_FH_ during DSA generation.

γδ T cells are innate-like lymphoid cells, which respond to “innate” signals such as cytokines, which have been shown to potentiate γδ TCR-induced activation [42] and proliferation [43]. However, the addition interleukin (IL)-18 or a combination of IL-2 and IL-15 during the culture with the beads coated with anti-CD3 and anti-CD28 mAbs had no impact on the expression profiles of CD69, CXCR5 or CD40L (Supplementary Figures S2A–D). Finally, to rule out the possibility that the PBMC may not recapitulate the features of cells in secondary lymphoid organs, we performed the same analyses with human splenocytes and obtained exactly the same results (Supplementary Figures S3A–C).

To confirm these results at the functional level we set up a coculture model mimicking the interactions occurring between B and T_FH_ cells in the germinal center reaction. The canonical sequence is initiated by the binding of the (allo)antigen to surface BCR, which delivers the first signal of activation to B cells. This leads to the internalization of the antigen, which is then processed for presentation within the MHC-II molecules on B cell surface. These complexes are recognized by a cognate CD4^+^ αβ T cell, which in response to this TCR-mediated activation, delivers the costimulatory signal (signal 2) to B cell. The sum of these two signals drives B cell proliferation and differentiation into DSA-producing plasma cell [10, 11, 44, 45]. To mimic this complex process *in vitro* we had to overcome the barrier of antigen specificity and used two tricks: i) signal 1 was delivered with an anti-IgM mAb, which cross-linked the BCR and activated the B cell clones regardless of their specificity [13], and ii) allogeneic CD4^+^ αβ T cells were used in the coculture because ∼10% of the latter directly recognize allogeneic MHC-II molecules on B cell surface [46]. These coculture conditions (Figure 2G) lead to an efficient proliferation of B cells as assessed by the dilution of a proliferation dye (Figure 2H). The intensity of the T-B dialogue within the immune synapse was also appreciated based on the acquisition by B cells of surface molecules from the T cells with which they interacted [a process known as trogocytosis, [47]]. After 6 days of coculture with allogeneic CD4^+^ αβ T cells, ∼70% of B cells expressed CD4 and CD3 (Figure 2I). As compared with B cells cocultured with allogeneic CD4^+^ αβ T cells, those cocultured with allogeneic γδ T cells (Figure 2G) did not proliferate (Figure 2H) and no trogocytosis was observed in the latter condition (Figure 2I), demonstrating that γδ T cells are not able to interact with MHC-II molecules expressed at the surface of B cells, and that BCR-activated B cells do not upregulate any surface antigen capable of activating γδ T cells. Thus, we concluded that γδ T cells are not able to perform “T_FH_-like” functions.

### Evaluation of T_FH_-Helper Function of Human γδ T Cells

**FIGURE 3:**
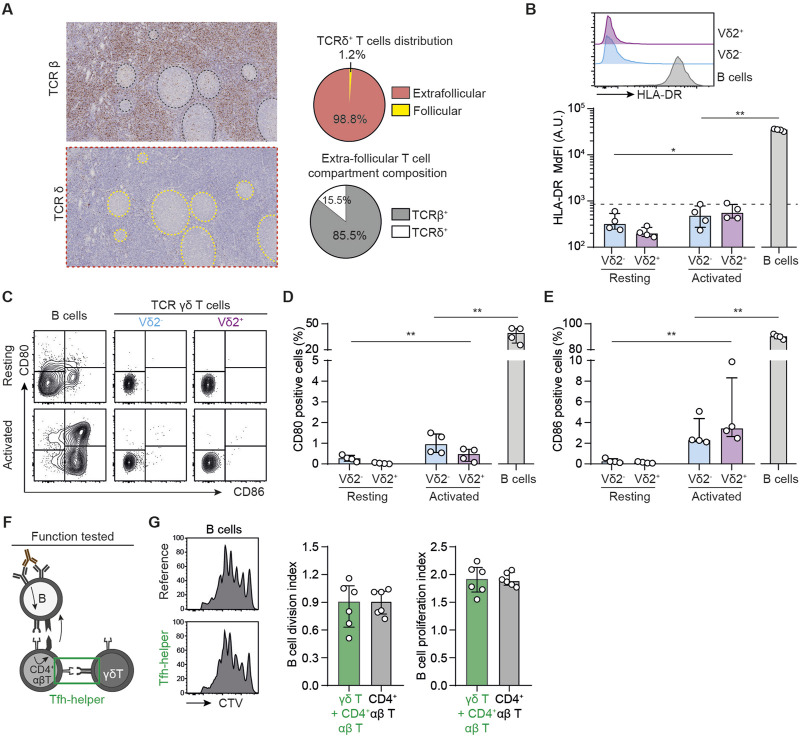
T_FH_-helper function of human γδ T cells **(A)** Left: immunohistochemical sections of human lymph node, stained for TCRβ (upper thumbnail) and TCRδ (lower thumbnail). Right: the density of TCRβ^+^ and TCRδ^+^ cells in the follicles were quantified by computer-assisted morphometry. Pie-chart representing the distribution of TCRδ^+^ T cells (up) and the proportion of TCRβ^+^ and TCRδ^+^ cells among extra-follicular T cells (down). **(B–E)** PBMCs were cultured in the presence or absence of beads coated with anti-CD3 and anti-CD28 mAbs. **(B)** Up: Representative histograms for the expression of HLA-DR in Vδ2^+^ (up, purple), Vδ2^−^ (middle, blue) γδ T cells or control B cells (down, grey). Down: individual MdFI values for HLA-DR^+^ cells. The dashed line represents the negative control. **(C)** Representative flow cytometry profiles for the expression of CD80 and CD86 in resting (upper row) and activated (lower row) T cells. **(D, E)** Individual values for percentages of **(D)** CD80^+^ and **(E)** CD86^+^ cells. **(F, G)** BCR-primed human B cells were cocultured with allogeneic CD4^+^ T in the presence or absence of syngeneic γδ T cells. **(F)** Schematic representation of the experiment. **(G)** The percentage of divided cells among alive B cells was evaluated by flow cytometry. Left: Representative histograms. Middle: individual B cell division index values. Right: individual B cell proliferation index values. Data are presented as median ± IQR. Data were analyzed by Mann-Whitney test when two groups were compared, Kruskal-Wallis test when more than two groups were compared, and two-way ANOVA when there was a within-group comparison between two different conditions. *P < 0.05 and **P < 0.01.

If γδ T cells are not able to directly help B cells for the production of DSA, they could however indirectly act by supporting T_FH_ cells. This hypothesis is suggesed by i) the fact that the vast majority of γδ T cells are located outside germinal centers, in the T cell area of secondary lymphoid organs, in which they form a network intertwined with that of the αβ T cells (Figure 3A), and ii) previously published studies showing that γδ T cells can present antigenic peptides within MHC-II [30, 48] and promote the differentiation of murine CD4^+^ T cells into T_FH_ [30].

To test this hypothesis, we performed a new set of experiments using the same *in vitro* model as described in the previous paragraph except that B cells, which are antigen-presenting cells (APC), were used as reference. To assess the ability of γδ T cells to present antigens, we first measured their expression of HLA-DR. Neither Vδ2^+^ nor Vδ2^−^ cells expressed HLA-DR in baseline conditions and if this expression was slightly increased after activation, the MdFI of HLA-DR remained logarithmically lower than that observed in B cells (Figure 3B). The same was proven true for the expression of costimulatory molecules CD80 and CD86 by the 2 subsets of γδ T cells (Figures 3C–E). These results remained unchanged when cytokines were added to the cultures (Supplementary Figures S2E–G) or when experiments were conducted with human splenocytes instead of PBMC (Supplementary Figures S3D–F).

Finally, to test the ability of γδ T cells to act as APCs in a more functional assay, we replicated the coculture described in the previous paragraph, adding or not γδ T cells to the reference condition (Figure 3F). The presence of γδ T cells in the coculture did not increase the number of dividing B cells (B cell division index, Figure 3G) or the number of divisions of those dividing B cells (B cell proliferation index, Figure 3G) as compared with the reference condition.

Overall, these results suggest that γδ T cells are unable to support CD4^+^ T_FH_ function.

### Validation of the *In Vitro* Findings in the Murine Model of Heart Transplantation

**FIGURE 4:**
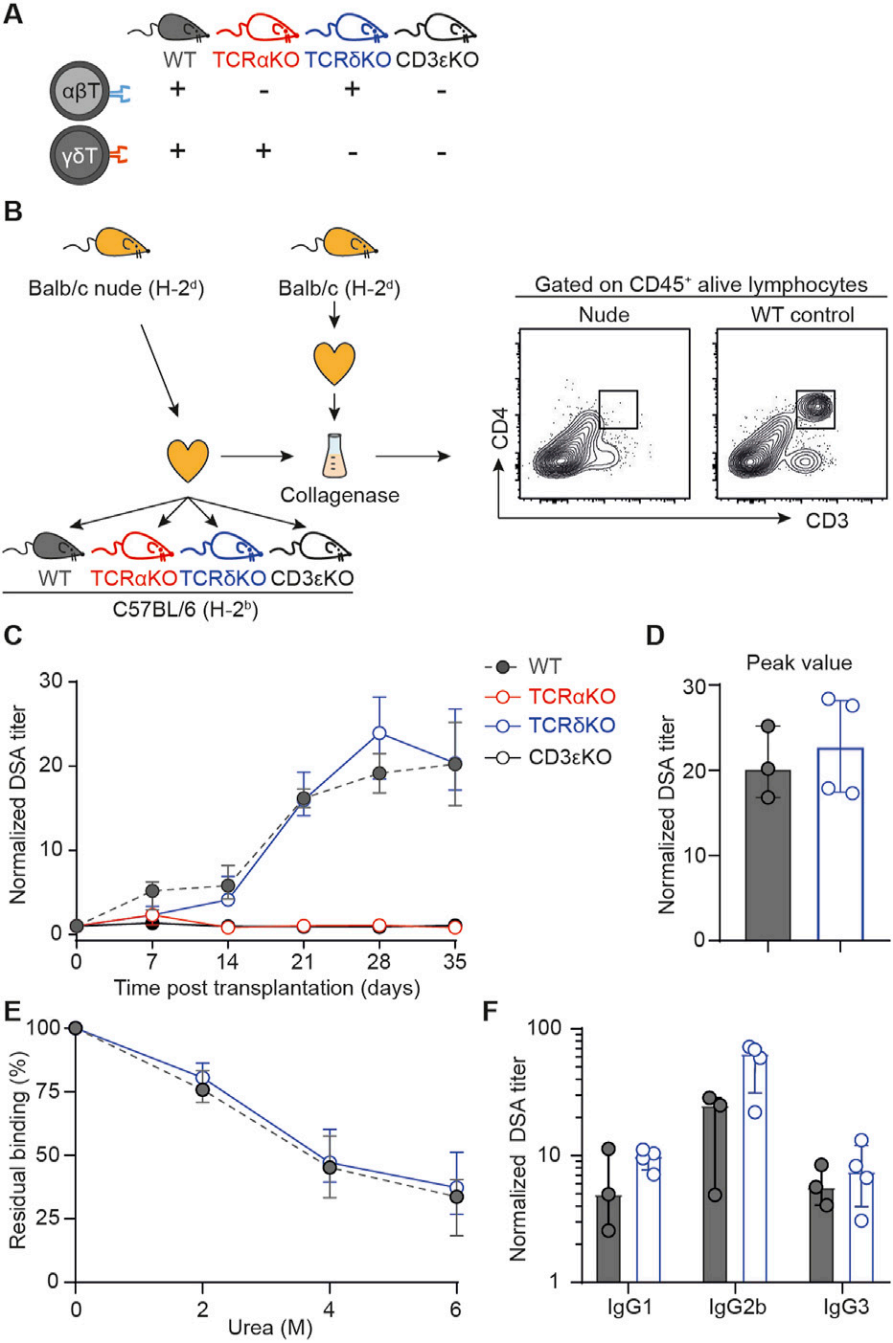
Assessment of the role of γδ T cells in a mouse model of heart transplantation **(A)** Schematic representation of the mouse strains used as recipient of an allogeneic heart transplant regarding αβ and γδ T cells compartments. **(B)** Presentation of the mouse model. Allogeneic Balb/c nude (H-2^d^) hearts were transplanted to wild-type (WT), TCRαKO, TCRδKO or CD3εKO C57BL/6 (H-2^b^) recipient mice. Results are from one experiment. Heart grafts were harvested from Balb/c nude and Balb/c WT donors and digested with collagenase. Heart graft cell suspensions were analyzed by flow cytometry. Representative flow cytometry profiles are shown. **(C)** Evolution of normalized DSA titers in the circulation of recipients is shown for wild-type (grey, n = 3), TCRαKO (red, n = 4), TCRδKO (blue, n = 4) and CD3εKO (black, n = 5) C57BL/6 mice. **(D)** DSA titers were compared at the peak of the response between wild-type (grey, n = 3) and TCRδKO (blue, n = 4) C57BL/6 mice. **(E)** The avidity of DSA produced by wild type (grey, n = 2) and TCRδKO (blue, n = 4) C57BL/6 recipients were compared at day 28 by assessing the stability of DSA binding to Balb/c splenocytes in the presence of increasing concentrations of urea used as chaotropic agent. **(F)** DSA isotypes were tested at the peak of the response for wild-type (grey, n = 2) and TCRδKO (blue, n = 4) C57BL/6 mice. Data are presented as mean ± SD. Data are presented as median ± IQR. Abbreviations: TCR, T-cell receptor; WT, wild-type.

The clinical study as well as the *in vitro* findings strongly indicate that γδ T cells are not involved in the generation of DSA after transplantation. To validate these results definitively, we initiated a last round of experiments using an *in vivo* experimental murine model of heterotopic heart transplantation. Different recipient mice, all on C57BL/6 (H-2^b^) background, were used: i) wild-type mice (presence of both αβ and γδ T cells; positive controls), ii) TCRαKO mice (absence of αβ T cells), iii) TCRδKO mice [absence of γδ T cells, [33]], and iv) CD3εKO mice (absence of both αβ and γδ T cells, negative controls); Figure 4A. The phenotypic characteristics of the 4 recipient mice strains were controlled before transplantation by flow cytometry (Supplementary Figures S4A–C). TCRαKO mice had no αβ T lymphocytes in the periphery but a normal γδ T cells count. They were therefore used to test the T_FH_-like function hypothesis of γδ T cells. Conversely, TCRδKO mice, which are devoid of γδ T cells but have a normal number of αβ T lymphocytes, were used to test whether γδ T cells are endowed with T_FH_-helper function. Finally, we also controlled that the 3 mutant mouse strains had similar B cell counts as compared with wild type mice (Supplementary Figure S4B).

These mice were used as recipients of a fully mismatched heart graft harvested from nude Balb/c (H-2^d^) donors. Nude donors were used because, in contrast with heart coming from wild type Balb/c, grafts from athymic mice did not contain T cells (Figure 4B). This trick allowed to completely suppress the inverted direct pathway, in which passenger T cells from donor origin interact with recipient’s B cells to trigger the generation of DSA [13, 14].

As expected, wild-type (positive control) mice generated DSA, which became detectable as early as 7 days post-transplantation and peaked at day 28 (Figure 4C). In contrast, neither CD3εKO (negative controls), nor TCRαKO mice developed detectable DSA after allogeneic heart transplantation. This total lack of DSA response is not explained by a defect in B-cell functionality in recipient mice as both mice strains generated normal antibody titers after immunization with the thymo-independent model antigen 4-hydroxy-3-nitrophenyl acetyl(NP)-Dextran (Supplementary Figure S4D). This result demonstrates that γδ T cells are unable to act as surrogate T_FH_ to generate DSA after transplantation.

TCRδKO mice produced DSA with similar kinetics (Figure 4C) and their response reached the same titer at peak as wild-type controls (Figure 4D). Furthermore, neither the affinity maturation, evaluated by the residual binding capacity of DSA in the presence of a chaotropic agent (urea, Figure 4E), nor the class switching of the DSA response appeared to be affected by the absence of γδ T cells (Figure 4F). These results demonstrate that γδ T cells are unable to provide help to T_FH_ for priming and during ongoing germinal center responses.

## Discussion

In this translational study, we demonstrated that γδ T cells can neither interact directly and serve as surrogate T_FH_ cells for allospecific B cells, nor act indirectly by supporting CD4^+^ αβ T_FH_. How can we reconcile these findings with recent publications, which suggested that γδ T cells could also be involved in adaptive immune responses [27], including the generation of antibodies [28–30]?

First, a single study has identified Vδ2^+^Vγ9^+^ T cells expressing CXCR5, CD40L, and ICOS in human inflamed tonsils and demonstrated that these cells could serve as surrogate T_FH_ *in vitro* [49]. However, i) alloantigens are not drained to the tonsils after transplantation, ii) T_FH_-like γδ T cells were not found in the periphery [49] and iii) finally, we could not reproduce these results with activated human peripheral Vδ2^+^ T cells (Figure 2). Another team has reported that mice lacking αβ T cells can produce autoantibodies [28, 50]. However, in contrast with alloantigens, which are exclusively proteins, many autoantigens are not. It is for instance the case of nucleic acids, which trigger the joint ligation of the BCR and TLRs in B cells [51]. It can thus be hypothesized that TLR signaling induces the expression of stress antigens [52] that would enable the interaction of autoreactive B cells with γδ T cells, which is not the case for alloantigens.

Second, a recently published study reported a role of γδ T cells in the response to an exogenous antigen, through the induction of T_FH_ differentiation [30]. However, in this work, γδ T cells were involved in the response against ovalbumin only when the antigen was adjuvanted with CFA (and not with alum), meaning that T-cell helping capacity is highly context dependent. In this regard, our data, in particular those obtained in the murine heart transplantation model, demonstrate that organ transplantation is not an immunological context allowing γδ T cells to prime CD4^+^ αβ T_FH_ cells (Figure 4). Another study has demonstrated the ability of γδ T cells to present antigens to CD4^+^ T cells *in vitro* [48]. This ability was limited to γδ T cells from tonsils, and our results show that this is not the case in the blood or the spleen. It has also been suggested that γδ T cells can perform antigen cross-presentation, but this mechanism is restricted to antigen presentation via HLA class I molecules and therefore to CD8^+^ T cell activation [53].

Finally, our findings could have significant implications for cell therapy. γδ T cells play a crucial role in protecting KTRs from both CMV infection and cancer [21, 54]. Employing cell therapy based on the adoptive transfer of γδ T cells could offer a promising avenue for addressing these major complications associated with therapeutic immunosuppression. This innovative approach offers two distinct advantages over conventional methods reliant on αβ T cells. Firstly, γδ T cells circumvent the obstacles posed by MHC compatibility, a common barrier to the use of αβ T cells. Secondly, our research underscores that γδ T cell-based therapy does not trigger the emergence of *de novo* DSA in KTRs, which poses a substantial risk to graft long-term viability.

In conclusion, our study demonstrates that γδ T cells are unable to function as surrogate T_FH_ cells or support CD4^+^ αβ T_FH_ during DSA generation, which remain therefore unfazed by their absence.

## Data Availability

The raw data supporting the conclusion of this article will be made available by the authors, without undue reservation.

## Footnotes

1
http://www.sfr-biosciences.fr/plateformes/animal-sciences/AniRA-PBES

2
https://www.R-project.org
