# Supplementary material for: γδ T Cells’ Role in Donor-Specific Antibody Generation: Insights From Transplant Recipients and Experimental Models
Source: Transpl Int. 2025 Jan 29;38:12859. doi: 10.3389/ti.2025.12859 (PMC11815947; doi:10.3389/ti.2025.12859)
Supplement: Supplementary file 1 [file DataSheet1.docx]

# Supplementary materials

**Supplementary methods**

## Flow cytometry analyses for the monitoring of γδ T cells

Immunophenotypic characterization was carried out on 100 µl anticoagulated whole blood. At least 5000 total lymphocytes were acquired after staining with anti-CD45, antipan-δ (clone IMMU 510; Beckman Coulter, Krefeld, Germany), and anti-TCR Vδ2 (clone 15D; Thermo Fisher Scientific, Rockford, IL). Percentages of cell populations were obtained using the CELLQUEST software (BD Bioscience), and absolute counts of lymphocytes were obtained using the Single–Platform Lyse/No–Wash Trucount (BD Bioscience).

## γδ T cell activation

Human Peripheral Blood Mononuclear Cells (PBMCs) were collected from the French National Blood Service (Etablissement Français du Sang, EFS) and isolated by centrifugation on a Ficoll density gradient.

After activation and removal of the Dynabeads, cells were incubated with the following antibodies: CD3 (UCHT1, BD Biosciences), CD4 (SK3, BD Biosciences), TCRγδ (REA-591, Miltenyi Biotec), Vδ2 (REA-771, Miltenyi Biotec), CD19 (HIB19, BD Biosciences), CXCR5 (RF8B2, BD Biosciences), CD69 (FN50, BD Biosciences), MHC-II (G46-6, BD Biosciences), CD80 (2D10, Biolegend), CD86 (FUN-1, BD Biosciences), and a fixable viability dye (ThermoFisher Scientific).

## Flow cytometry

After staining, samples were acquired on a BD LSRFortessa flow cytometer (BD Biosciences). Data were analyzed with FlowJo software (Tree Star).

## Lymph node histology

The samples analyzed by histology were normal peripheral lymph nodes. Staining of formalin-fixed paraffin-embedded (FFPE) sections was performed by automated immunohistochemistry (LEICA BOND-III, Leica Biosystems) using anti-human TCRβ (anti-T-cell receptor [TCR]β antibody; clone G11; Santa Cruz Biotechnology) and TCRδ (anti–T-cell receptor [TCR]δ antibody; clone H41; Santa Cruz Biotechnology) mAbs. Computer-assisted morphometric quantifications were performed using FIJI software ^1^.

## Characterization of the immune phenotype of the different mouse strains

Before staining, murine cells from spleen, lymph nodes, heart or blood were incubated with a blocking anti-mouse Fc receptor antibody (2.4G2, home-made hybridoma). Cells were then incubated at 4°C with fluorescent antibodies: CD3 (145-2C11), CD19 (1D3), TCRβ (H57-597, BD Biosciences) and TCRδ (GL3), all from BD Biosciences. Before analysis by flow cytometry, DAPI (4′,6-diamidino-2-phenylindole dihydrochloride, Sigma-Aldrich) was added to the cell suspension to exclude dead cells. Sample acquisitions were made on a BD LSR II flow cytometer (BD Biosciences).

## Mice

Wild-type C57BL/6 (H-2^b^) mice and wild-type or nude Balb/c (H-2^d^) mice were purchased from Charles River Laboratories (Saint Germain sur l’Arbresle, France). TCRαKO ^2^ mice were obtained from the Centre de Distribution, Typage et Archivage animal (Orléans, France). TCRδKO ^3^ mice were provided by B. Malissen. CD3εKO mice were purchased from The Jackson Laboratory (Bar Harbor, ME, USA). TCRαKO, TCRδKO and CD3εKO mice were on C57BL/6 genetic background.

All mice were maintained under EOPS (Exemption of Specific Pathogenic Organisms) conditions in our animal facility: Plateau de Biologie Expérimentale de la Souris (http://www.sfr-biosciences.fr/plateformes/animal-sciences/AniRA-PBES; Lyon, France).

## Functional evaluation of the B cell compartment of the different mouse strains

Mice were immunized intraperitoneally with 200 µg NP-Dextran. Sera were tested for IgM anti-NP antibodies. Maxisorp plates (Nunc) were coated with NP 23-conjugated BSA. Serially diluted serum samples were added for 1 h 30 at room temperature. NP-specific antibodies were detected with alkaline phosphatase conjugated goat anti-mouse IgM Abs (1/2,000 dilution) followed by phosphatase substrate (Sigma-Aldrich). The plates were read at 405 nm/490 nm with an automatic reader (Zeiss VERSAmax). We used standard curves to convert OD to concentration using a four-parameter logistic equation (Softmax Pro 5.3 software; Molecular Devices).

***Characterisation of the cellular content of the transplants***

For the characterization of the cellular content of the transplants, hearts harvested from Balb/c wild-type and nude mice were sequentially digested in 10mL collagenase type II (500 UI/mL, Worthington, Lakewood, NJ), then in 5 mL collagenase/dispase (1mg/mL, Roche Diagnostics), for 30 minutes at 37°C each time. Single cell suspensions were incubated with a blocking anti-mouse Fc receptor antibody (clone 2.4G2) for 20 minutes at 4°C and then with relevant fluorescent monoclonal antibodies: CD45 (clone 30-F11, BioLegend), CD3 (clone 145-2C11, BD Biosciences) and CD4 (clone RM4-4, BD Biosciences) for 10 minutes at 4°C. Before acquisition, 0.1μg/ml 4’,6-diamidino-2-phenylindole (DAPI) was added to the cell suspension to stain the dead cells. Sample acquisitions were made on an LSR II flow cytometer (BD Biosciences) and analyses were performed with FlowJo software version 10.0.8r1 (Tree Star Inc, Ashland, OR).

## Determination of DSA titer and avidity

DSA titers were determined as follows: briefly, Balb/c CD4^+^ T cells were incubated with the sera of sensitized recipients. Binding of DSA to Balb/c cells was revealed using an anti-kappa light chain (187.1, BD Biosciences), anti-IgG1 (A85-1, BD Biosciences), anti-IgG2b (SouthernBiotech) or anti-IgG3 (R40-82, BD Biosciences) secondary antibody. Syngeneic C57BL/6 CD4^+^ T cells were used as controls. The titer of anti-donor antibodies at each time point (dx) was calculated with the following formula: normalized DSA titer = [MFI Balb/c (dx)/ MFI C57BL/6 (dx)]/ [MFI Balb/c (d0)/ MFI C57BL/6 (d0)]. Avidity of DSA was estimated by measuring the stability of preformed antigen-antibody complexes in the presence of increasing concentrations of a chaotropic agent (urea).

**Supplementary Table 1. Baseline kidney recipients characteristics**

| N (%) or mean±SD | Whole cohort  n=331 | De novo DSA within 10 years  n=62 | No DSA  n=269 |
| --- | --- | --- | --- |
| **Age at time of transplantation (y)** | 50±15 | 45±18 | 51±14 |
| **Male** | 223 (67%)) | 38 (61%) | 185 (69%) |
| **Blood group**  O  A  B  AB | 132 (40%)  157 (47%)  26 (8%)  16 (5%) | 21 (34%)  34 (55%)  4 (6%)  3 (5%) | 111 (41%)  123 (46%)  22 (8%)  13 (5%) |
| **Cause of renal failure**  Glomerulonephritis  Diabetes mellitus  Vascular  Hereditary  Uropathy  Others | 82 (25%)  27 (8%)  16 (5%)  73 (22%)  30 (9%)  103 (31%) | 20 (32%)  4 (6%)  3 (5%)  10 (16%)  6 (10%)  19 (31%) | 62 (23%)  23 (9%)  13 (5%)  63 (23%)  24 (9%)  84 (31%) |
| **Donor**  Age  Living  Deceased | 48±17  23 (7%)  308 (93%) | 46±20  3 (5%)  59 (95%) | 48±16  20 (7%)  249 (93%) |
| **Transplantation condition**  First transplantation  No. of HLA A/B/DR/DQ mismatches | 320 (97%)  4.9±1.5 | 59 (95%)  5.5±1.5 | 261 (97%)  4.8±1.5 |
| **CMV status**  D^-^/R^-^  D^+^/R^-^  R^+^ | 79 (24%)  75 (23%)  177 (53%) | 14 (23%)  11 (18%)  37 (60%) | 65 (24%)  64 (24%)  140 (52%) |
| CMV < 2 years  Disease  DNAemia | 48 (15%)  92 (28%) | 10 (16%)  18 (29%) | 38 (14%)  74 (28%) |

Abbreviations: DSA, donor-specific antibodies; y, years; No., number; CMV, cytomegalovirus; D/R, donor/recipient.

**

**

***Supplementary Figure 1. Flow-chart of the study***

Abbreviations: KTR: kidney transplant recipients; mAb: monoclonal antibody; MMF: mycophenolate mofetil; DSA: donor-specific antibody.

**
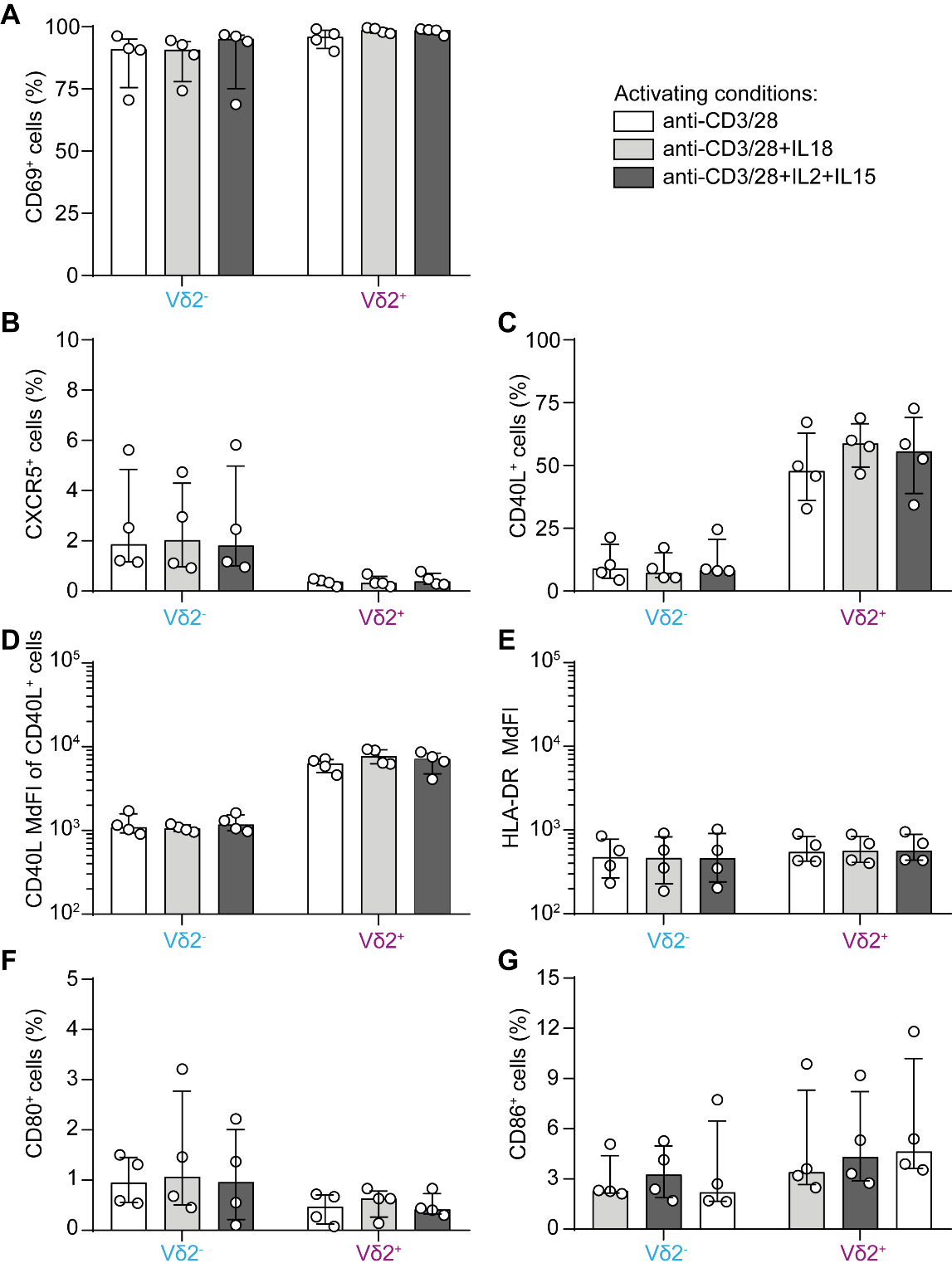
**

***Supplementary Figure 2. Comparison of different activation cocktails***

PBMCs were activated with beads coated with anti-CD3 and anti-CD28 mAbs, in the presence or absence of IL-18 or IL-2+IL-15.

**(A to C)** Individual values for percentages of **(A)** CD69^+^, **(B)** CXCR5^+^ and **(C)** CD40L^+^ cells.

**(D and E)** Individual MdFI values for **(D)** CD40L^+^ and **(E)** HLA-DR^+^ cells.

**(F and G)** Individual values for percentages of **(F)** CD80^+^ and **(G)** CD86^+^ cells.


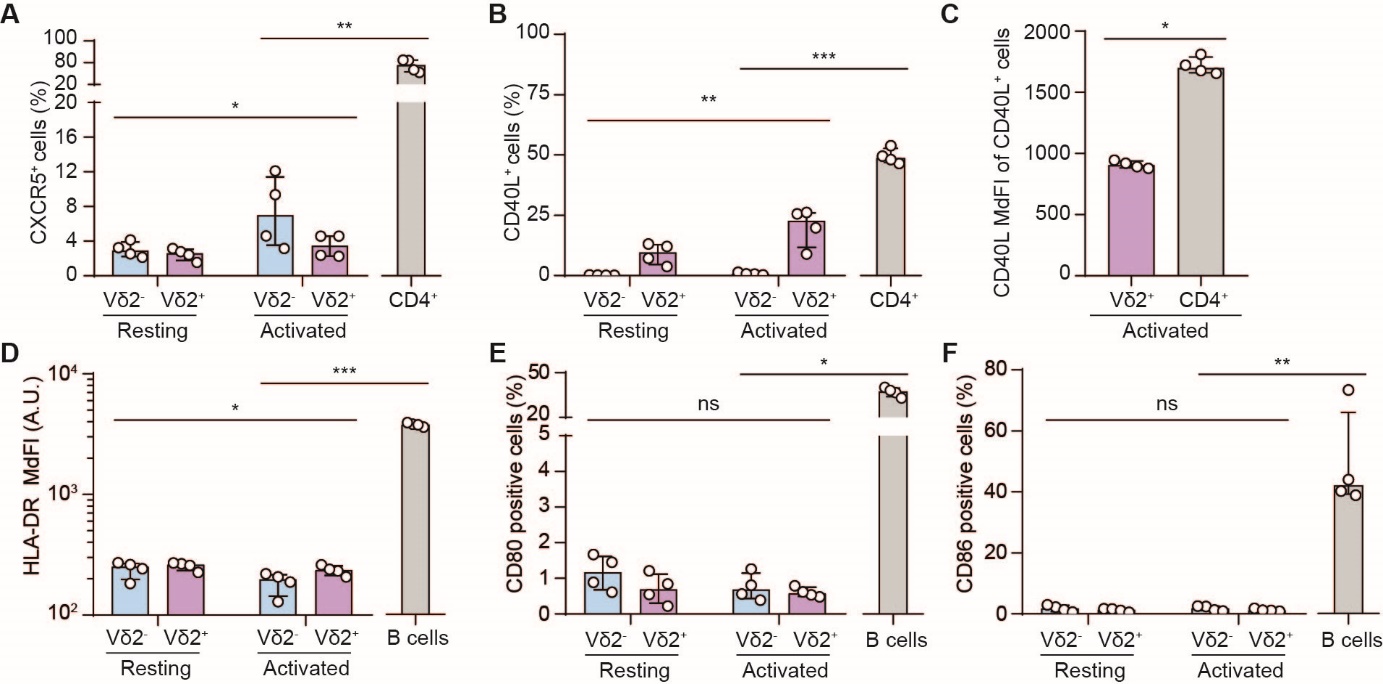


***Supplementary Figure 3. T_FH_-like and T_FH_-helper function of human γδ T cells***

Human splenocytes were cultured in the presence or absence of beads coated with anti-CD3 and anti-CD28 mAbs, together with IL-2 and IL-18.

**(A-B)** Individual values for percentages of (A) CXCR5+ and (B) CD40L+ cells.

**(C)** Individual MdFI values for CD40L^+^ cells.

**(D)** Individual MdFI values for HLA-DR^+^ cells.

**(E-F)** Individual values for percentages of (E) CD80+ and (F) CD86+ cells.

**
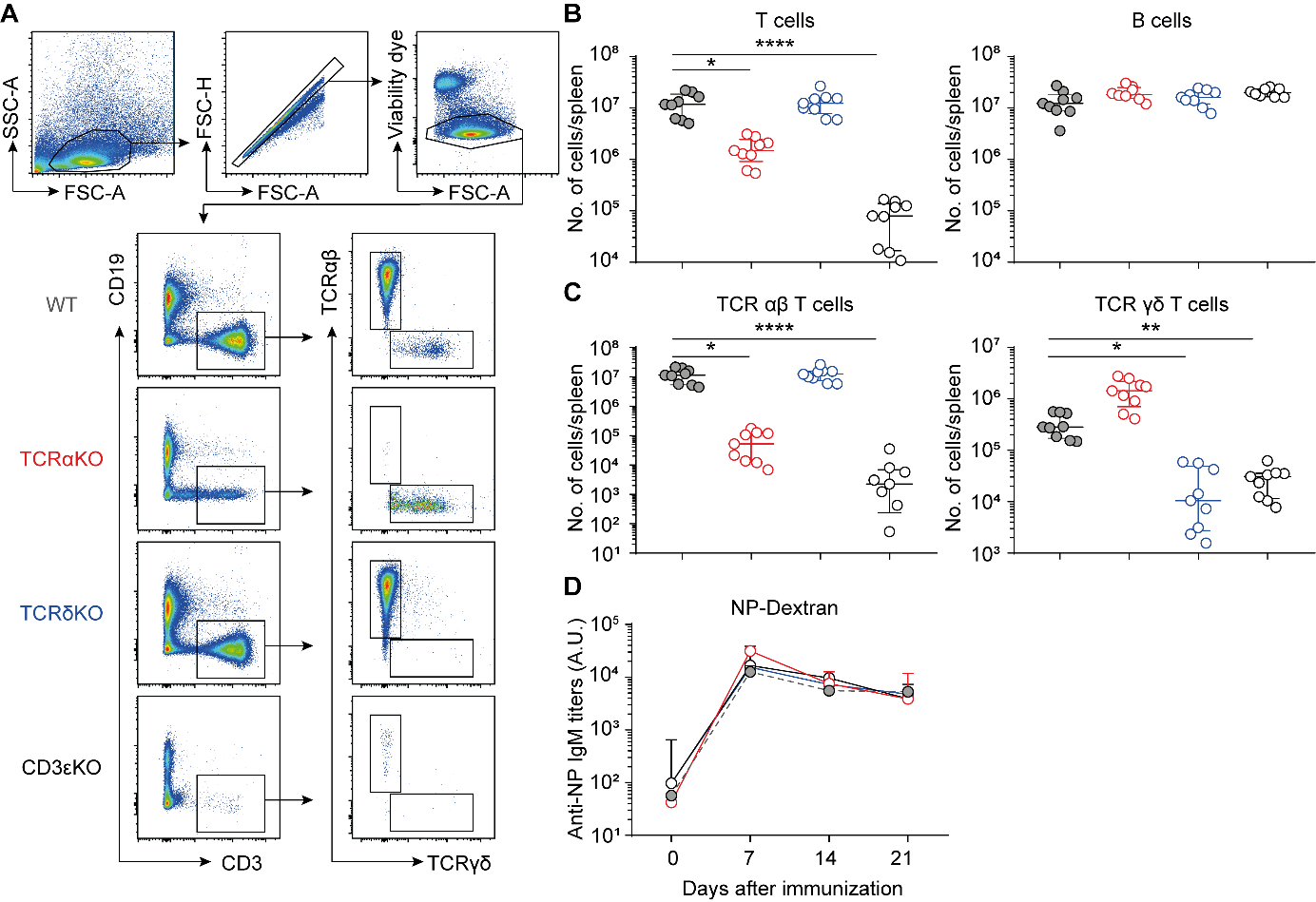
**

***Supplementary Figure 4. Phenotypic and functional assessment of the four murine strains***

**(A)** Flow cytometry gating strategy.

**(B)** Absolute number of T cells (left panel) and B cells (right panel) contained in the spleen of wild-type (WT, grey, n=9), TCRαKO (red, n=8), TCRδKO (blue, n=9) and CD3εKO (black, n=8) C57BL/6 mice.

**(C)** Absolute number of TCR αβ T cells (left panel) and TCR γδ T cells (right panel) contained in the spleen of wild-type (WT, grey, n=9), TCRαKO (red, n=8), TCRδKO (blue, n=9) and CD3εKO (black, n=8) C57BL/6 mice.

**(D)** Animals were immunized with the thymo-independent model antigen NP-Dextran and IgM titers were measured. Comparison over time of anti-NP antibody titres of wild-type C57BL/6 (grey, n=3), TCRαKO (red, n=3), TCRδKO (blue, n=3) and CD3εKO (black, n=3).

Data are presented as median ± IQR. Kruskal-Wallis test with Dunn’s multiple comparisons. *P<0.05; **P<0.01; ****P<0,0001.

Abbreviations:; TCR, T-cell receptor; No., number; WT, wild-type.

**Supplementary references**

1. Schindelin J, Arganda-Carreras I, Frise E, et al. Fiji: an open-source platform for biological-image analysis. *Nat Methods*. 2012;9(7):676-682. doi:10.1038/nmeth.2019

2. Mombaerts P, Clarke AR, Rudnicki MA, et al. Mutations in T-cell antigen receptor genes α and β block thymocyte development at different stages. *Nature*. 1992;360(6401):225-231. doi:10.1038/360225a0

3. Itohara S, Mombaerts P, Lafaille J, et al. T cell receptor δ gene mutant mice: Independent generation of αβ T cells and programmed rearrangements of γδ TCR genes. *Cell*. 1993;72(3):337-348. doi:10.1016/0092-8674(93)90112-4
